# Zika: the continuing threat

**DOI:** 10.2471/BLT.19.020119

**Published:** 2019-01-01

## Abstract

The steep decline in Zika cases since 2016 has led to a perception that the threat posed by the virus has diminished. Recent outbreaks and global spread underline the need for continued vigilance. Tatum Anderson and Gary Humphreys report.

On 11 September 2018, a 78-year-old resident of Shastri Nagar, an area to the north of Jaipur in Rajasthan, India, checked into the Sawai Man Singh hospital reporting joint pain and weakness.

Tests for dengue and influenza came back negative. Samples of the woman’s blood were sent to the National Institute of Virology in Pune, Maharashtra state, where it was determined that she was infected with the Zika virus.

State health authorities were notified and initiated a response that included boosting surveillance and control efforts in the Shastri Nagar area.

According to Dr Neena Valecha, Director of the National Institute for Malaria Research in New Delhi, vector surveillance in and around Shastri Nagar resulted in about 200 *Aedes* a*egypti* mosquitos being caught, three of which tested positive for Zika. 

“This is the first time the Zika virus has been isolated in the mosquito population in India, and it suggests that the disease is being transmitted locally rather than being brought in from outside,” she says.

A second outbreak, located in the neighbouring state of Madhya Pradesh, also started in November. 

As of 20 November 2018, 160 people had been confirmed to be infected with Zika virus in the Jaipur outbreak, including 63 pregnant women. In the Madhya Pradesh outbreak, 120 people, including 34 pregnant women, had been confirmed infected.

“This is the first time the Zika virus has been isolated in the mosquito population in India.”

Neena Valecha

These outbreaks are just the latest reminders of two important facts. First, the risk of becoming infected with the Zika virus is global. The Zika virus is present in more than 84 countries. Second, transmission is ongoing and the virus can be expected to spread.

Transmitted primarily by *Aedes* mosquitoes, the Zika virus was first reported in humans in 1952. It was not associated with severe disease until an outbreak in French Polynesia in 2013–2014 and to this day only around 20% of people infected suffer any symptoms at all. Most of those infected experience mild symptoms such as fever, rash, conjunctivitis, muscle and joint pain, or headache.

Zika infection can, however, be followed by Guillain–Barré syndrome, a serious neurological condition in which the immune system attacks the peripheral nerves, but what has put the virus under the spotlight in recent years, most is its association with birth defects.

Zika infections in pregnant women can be transmitted to the developing fetus and can cause severe malformations that collectively make up congenital Zika syndrome: microcephaly (when the baby’s brain does not develop properly, resulting in a small head), limb contractures and eye abnormalities.

The 2015–2016 outbreak in the Region of the Americas brought the Zika virus, until then a little known viral infection, to the fore, revealing unknown complications and triggering a public health emergency.

At the height of the 2016 emergency, 216 207 probable cases of acute Zika virus disease were reported in Brazil, the focus of the epidemic, and an estimated 8604 babies were born with malformations.

In response, on 1 February 2016, the World Health Organization (WHO) declared a Public Health Emergency of International Concern, prompting a comprehensive response, including the development of surveillance networks, vector-control measures and risk communication strategies.

WHO also encouraged the development of diagnostic technologies and included Zika among the list of priority diseases targeted by WHO’s global strategy and preparedness plan, designed to facilitate research and development during epidemics, called the R&D Blueprint.

But just as the international response was ramping up, Zika cases started to decline globally.

“The most recent available data show that there has been a substantial decline in the number of reports of suspected cases of Zika in Brazil since 2016,” says Dr Márcio Henrique de Oliveira Garcia at the Secretariat of Health Surveillance, Ministry of Health of Brazil, who notes that there have been similar declines throughout the Americas.

Following this decline, WHO declared the end of the Public Health Emergency of International Concern in November 2016.

For Dr David Heymann, chair of the International Health Regulations Emergency Committee on the Zika Virus, which advises WHO on its Zika response, the decision reflected both the evolving epidemic and the response that had been initiated.

“Bringing the public health emergency to a close was recommended by the emergency committee to transition the program from an emergency response to a long-term commitment for prevention and control,” he says.

Three months later, the then Director–General of WHO, Dr Margaret Chan, said that WHO and affected countries needed to manage Zika not on an emergency footing, but in the same way they respond to other established epidemic-prone pathogens, like dengue and chikungunya, which in her words “ebb and flow in recurring waves of infection.”

The tidal metaphor raises compelling questions. Why did Zika ebb? Where and how will it re-emerge?

For Dr Eve Lackritz, the Zika taskforce lead in the high-threat pathogens unit of WHO’s Health Emergencies Programme, the Zika epidemic likely waned due to an increase in population-level immunity. The more that populations have been exposed to the virus, the more they have developed immunity, hence the decline in cases.

Answering the second question is harder. Some epidemiologists have suggested that populations with no previous exposure to the virus are most likely to experience outbreaks in the future. Lackritz points out that even in areas severely affected by the epidemic, there remain large numbers of people who were not exposed and therefore have not developed immunity.

“Smaller outbreaks continue to occur in the Americas and elsewhere as infection spreads to pockets that had previously been unaffected and where the population remains susceptible,” she says, pointing to ongoing case reports. 

Evidence of endemic transmission has been reported in Indonesia, Lao People's Democratic Republic, Singapore, Thailand and Viet Nam. “Transmission is likely ongoing, but remains poorly understood,” she says, emphasizing the need to strengthen surveillance systems worldwide to improve our understanding of the extent and patterns of Zika virus transmission.

“Evidence of the global spread of the epidemic (Brazilian) strain of Zika virus continues to accumulate.”

Eve Lackritz

A question of considerable importance for Valecha, is whether new outbreaks in India and elsewhere will result in cases of congenital Zika syndrome. Here too, the picture is unclear.

In addition to congenital malformations caused by the Brazilian Zika strain, Lackritz points out there have also been babies born with microcephaly after their mothers were infected with the endemic Asian strain reported in Thailand and Viet Nam.

In addition, there is evidence that the Brazilian Zika strain is spreading. “Evidence of the global spread of the epidemic (Brazilian) strain of Zika virus continues to accumulate,” she says. “Angola, for example, has reported a cluster of microcephaly cases and recently identified the introduction of the Brazilian Zika strain.”

Are India’s Zika virus outbreaks part of the pattern of smaller, localized outbreaks or the beginning of something bigger?

Prior to the current outbreaks in Rajasthan and Madhya Pradesh, there had been only four confirmed cases of Zika virus infection in the entire sub-continent, three of them in Ahmedabad Gujarat, which is also adjacent to Rajasthan. Low levels of population immunity might therefore be expected.

“The situation is a matter of concern,” says Valecha, but she is hopeful that the government’s vigorous response, which has included increased surveillance and monitoring and vector control efforts, will be sufficient to stop the outbreaks in their tracks or reduce transmission to a minimum.

However, she insists on the importance of focusing control efforts, ranging from larval control to personal protection measures, particularly in urban areas. “In the past, vector control has tended to focus on rural areas,” she says. “It is important to adapt vector control programmes to increasingly urbanized populations.” Lackritz concurs, noting that *Aedes* mosquitos thrive in urban environments, and breed in small collections of water in trash, used tyres and artificial containers.

Going forward, new vector control methods will give health authorities more options in the way they deal with Zika outbreaks. There are several in the pipeline, including Wolbachia-based biocontrol and OX513A transgenic mosquitoes.

A safe and effective Zika virus vaccine would also be helpful. Since 2016, research and development projects on 45 Zika vaccine candidates have been initiated. Several vaccine candidates are currently in phase 1 and 2 of clinical trials. However, many challenges remain, including the low number of cases occurring globally, which limits the ability of studies to evaluate the protective effects of vaccines in phase 3 trials.

Monitoring for congenital malformations will be an important focus of India’s Zika response in the coming months, as public health officials seek to determine the extent of the harm caused by the outbreaks. Says Valecha: “The true picture will only be visible when we follow these women who got the infection in the first trimester.”

**Figure:**
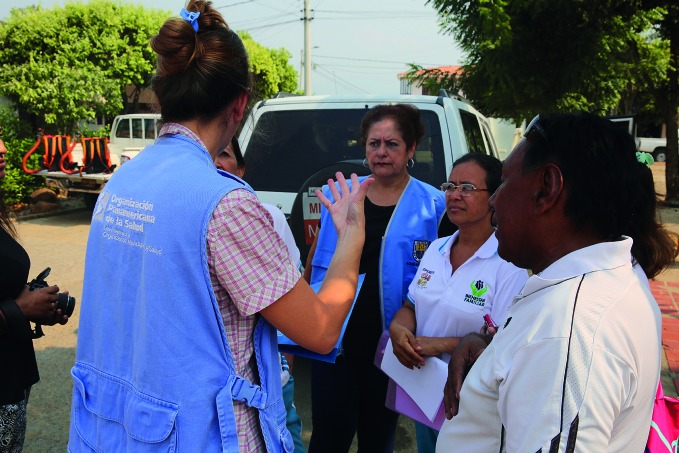
Team in Colombia raising awareness of how to prevent infection with the Zika virus.

© PAHO-WHO David Spitz

**Figure:**
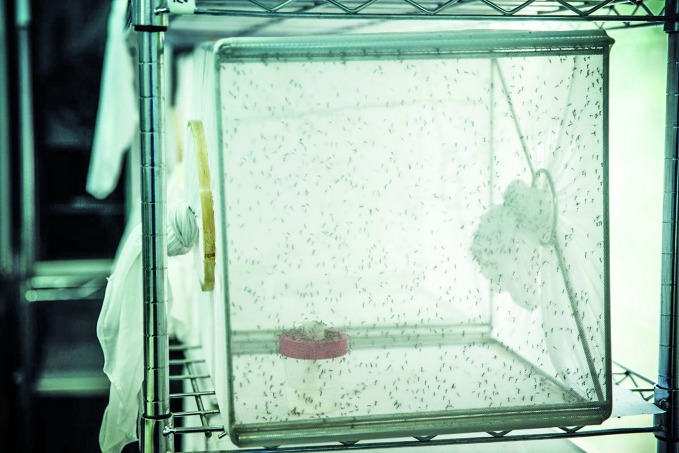
Individual mesh with 1000–1500 *Aedes aegypti* mosquitoes in the research lab at Gaiolão, Expansão Fiocruz as part of the World Mosquito Programme, Brazil.

©Gates Archive/Toni Greaves

